# Bound States in the Continuum and Long-Range Coupling of Polaritons in Hexagonal Boron Nitride Nanoresonators

**DOI:** 10.1021/acsphotonics.4c00358

**Published:** 2024-09-22

**Authors:** Harsh Gupta, Giacomo Venturi, Tatiana Contino, Eli Janzen, James H. Edgar, Francesco De Angelis, Andrea Toma, Antonio Ambrosio, Michele Tamagnone

**Affiliations:** † Istituto Italiano di Tecnologia, Via Morego 30, 16163 Genova, Italy; ‡ Dipartimento di Chimica e Chimica Industriale, Università Degli Studi di Genova, Via Balbi 5, 16126 Genova, Italy; § Center for Nano Science and Technology, Fondazione Istituto Italiano di Tecnologia, 20133 Milan, Italy; ∥ Dipartimento di Fisica, Politecnico Milano, Piazza Leonardo Da Vinci 32, Milano 20131, Italy; ⊥ Tim Taylor Department of Chemical Engineering, Kansas State University, Manhattan, Kansas 66506, United States

**Keywords:** phonon polaritons, nanoresonators, BICs, quality factor, miniaturization, nanophotonic devices

## Abstract

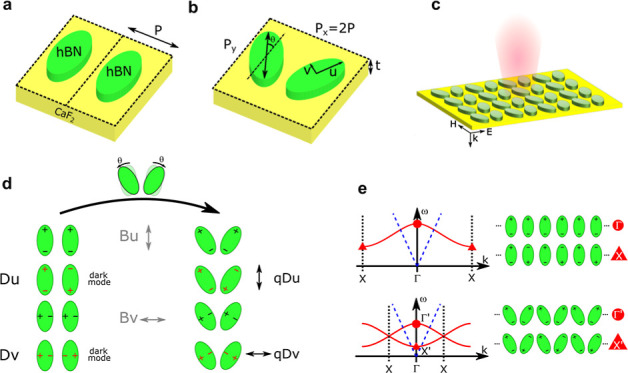

Bound states in the continuum (BICs) garnered significant interest for their potential to create new types of nanophotonic devices. Most prior demonstrations were based on arrays of dielectric resonators, which cannot be miniaturized beyond the diffraction limit, reducing the applicability of BICs for advanced functions. Here, we demonstrate BICs and quasi-BICs based on high-quality factor phonon-polariton resonances in isotopically pure h^11^BN and how these states can be supported by periodic arrays of nanoresonators with sizes much smaller than the wavelength. We theoretically illustrate how BICs emerge from the band structure of the arrays and verify both numerically and experimentally the presence of these states and enhanced quality factors. Furthermore, we identify and characterize simultaneously quasi-BICs and bright states. Our method can be generalized to create a large number of optical states and to tune their coupling with the environment, paving the way to miniaturized nanophotonic devices with more advanced functions.

## Introduction

Polaritons, a fascinating phenomenon in the realm of nanophotonics, offer a remarkable capability: subwavelength confinement of light at the nanoscale.^1−15^ This phenomenon arises from the strong coupling of photons with collective excitation in matter, such as electronic motion (plasmons) or vibrational modes (phonons).^5,9^ This marriage results in hybrid light-matter modes possessing unique properties, such as deeply subwavelength confinement due to the tight spatial localization of polariton modes, opening doors to a plethora of applications that benefit from their ability to beat the diffraction limit and to enhance light-matter interactions.

Examples include super-resolution imaging techniques,^2^ chemical and biological sensors and detectors with enhanced sensitivity,^1,16^ miniaturized nanophotonic devices, improved energy harvesting and photovoltaics, and metamaterials with novel optical properties.^17^

First described by von Neumann and Wigner in 1929,^18^ bound states in the continuum (BICs) have gained significant interest in photonics. These unique states remain bound without radiating, even if their energy levels allow it. Recent developments in BICs include their use in enhancing laser performance, improving sensing technologies,^19,20^ and enabling high-Q resonances in photonic crystals.^21^

Because of that, BICs have, in principle, an infinite lifetime and fine-tunability of frequency response based on the geometry of resonators, making a fundamental aspect that distinguishes them from the other resonant states that have a finite lifetime. However, the concept of BICs remained a theoretical curiosity because of its nonradiative nature until the concept of experimentally produced longer lifetime (not infinite) quasi-BIC states (q-BICs) with a little radiative nature was introduced in the 1970s^22^ when it was realized that they could have practical applications in areas such as photonics and microelectronics.^23^ In fact, BICs and q-BICs can occur for light as well, for instance, in nanophotonic resonators, where these states are essentially dark states and quasi-dark states, respectively.^23−31^ Most of the existing BIC systems are based on dielectric resonators,^25−27,32^ which, however, due to the diffraction limit, cannot be miniaturized beyond a fraction of wavelength. In this study, we investigate the existence of bound states in the continuum in arrays of high-quality phonon polariton resonators using isotopically pure hexagonal boron nitride (h^11^BN). We demonstrate here that BICs can be easily extended to hBN polaritons using structures that can be arbitrarily small, which opens many possibilities in terms of design complexity and allows for miniaturized devices with the ability to integrate multiple functionalities into a small footprint. We present here the conditions and mechanisms that give rise to these bound states in our system.

### Theory

BICs, facilitated by diverse dielectric, plasmonic, and phononic^33^ materials, mark a significant advancement in achieving high-Q phononic resonances.^34^ Dielectrics with high refractive index (such as silicon in the near-infrared) are generally used because they enable efficient light confinement and compact device integration, which makes them ideal for a wide range of applications.^29,35^ This characteristic renders them well-suited for various applications, enhancing our control over sound waves at a miniature scale.^36^ Not limited to dielectric, plasmonic BICs also have expanded our understanding and potential applications of high-Q resonances; for instance, Wang et al.^37^ demonstrated the enhancement of plasmonic BICs through dipole-coupled spontaneous emission, revealing novel mechanisms for achieving high-Q resonances in plasmonic systems.^37−40^ Additionally, Randerson et al.^39^ explored methods for controlling the generation of high-Q resonances in plasmonic BICs, offering insights into tailored design and optimization of nanophotonic devices.

**Figure 1:**
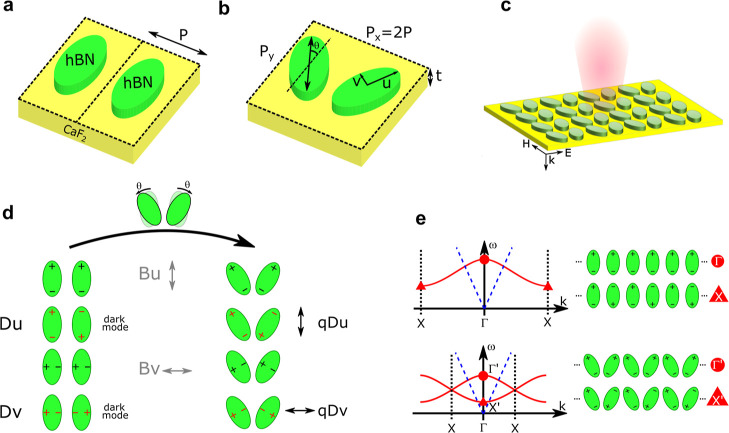
Unit cell and band structure of the BIC resonators. (a) A pair of symmetrical elliptical hBN resonators on the CaF_2_ substrate. Dash lines delimit the single-resonator unit cell. (b) A unit cell of the BIC system formed by two elliptical resonators rotated by θ = 25^°^ in opposite directions. For both panels, major axis 2*u* and minor axis 2*v* are 1.025 μm and 514 nm, respectively (quantities taken directly from the fabricated devices shown below), periodicity is *P* = 0.9 μm, *P*_*x*_ = *P*_*y*_ = 1.8 μm. hBN thickness is 50 nm. (c) Metasurface formed by an array of cells with incident and scattered light. (d) Bright and dark (BIC) modes supported by the unit cells have different resonances due to the coupling between resonators. When the rotation θ is introduced (in the limit of small θ), the previous BIC modes become q-BIC since they can now weakly couple to incident far-field light. However, the polarization coupled with far-field light must have orthogonal polarization with respect to the corresponding bright mode. (e) Schematic sketch of structures for symmetrical and symmetry-broken cells (the light cone is represented in dashed blue lines). For the symmetric system, the central point (Γ) represents the bright mode for normal incidence, while the band edge case (X) represents the dark mode, with dipoles having alternating signs. Breaking the symmetry halves the width of the band structure and folds it so that the previous X mode (X′) is now folded into the light cone and weakly couples to normal incidence light of orthogonal polarization. Coupling is responsible for the band structure to be different from a flat line and ultimately for the frequency shift between BIC and bright modes.

For this work, we directly adapted to polaritons one of the most common structures: an array of pairs of elliptical resonators where ellipses are rotated by a small angle θ in opposite directions (see Figure 1a,b). First, the elliptical polaritonic resonators are designed, and then the unit cell formed by the pair of ellipses is optimized. Elliptical-shaped resonators are used here because they are easier to fabricate experimentally and have been used successfully for BICs on other platforms. The first step was covered by previous works^1,5,14,15^ which considered disks, ribbons, and ellipses made of hBN. Here, the use of isotopically pure h^11^BN enhances the polariton propagation lengths about three times better than not enriched hBN, which significantly increases the quality factor of resonators without requiring any design alterations.^6,15^ Since hBN is a wideband insulator [with an indirect band gap of nearly (6.3 eV)],^41^ carrier losses are negligible, which preserves the quality of its natural hyperbolic phonon polaritons, making it a suitable candidate for this work. The uniaxial response is due to optical phonons with different dispersion for ion core oscillations parallel or perpendicular to the van der Waals layers. In the limit of small momentum, these optical phonons strongly couple with the photon field, creating a strong polaritonic response in two bands, the reststrahlen band 1 (RB1, Type-I, 764–820 cm^–1^) and 2 (RB2, Type II, 1360–1613 cm^–1^), created by out-of-plane and in-plane oscillations, respectively.

For the structures we considered, the momentum of the polaritons is much smaller than the reciprocal lattice of hBN. Thus, the nonlocality of phonon-polaritons can be neglected (compare the locality scale for instance^42^) and their optical response in the linear limit can be represented by a single oscillator per band in a simple Lorentz model. The permittivity tensor is, therefore, diagonal with ε_*xx*_ = ε_*yy*_ ≠ ε_*zz*_
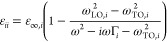
1where *i* ∈ {*x*,*y*,*z*}, ω = 2π*f* = 2π*c*/λ is the angular frequency, *c* is the speed of light, λ is the wavelength of the incident radiation, ε_∞,*i*_ is the high-frequency limit of the permittivity, Γ_i_ is the resonance line width, ω_LO,*i*_ and ω_TO,*i*_ are the upper and the lower frequency limits of the reststrahlen bands along *x*- and *z*-direction, respectively.^15^

Inside the reststrahlen bands, some of these entries are negative, which is the key to some of the properties of hBN polaritons, such as hyperbolicity. Here, however, hyperbolicity is not required, and we simply rely on the existence of surface waves that, when confined to the chosen elliptical shape, create the resonant modes. Because of this, the following steps can be extended to polaritons in other 2D and van der Waals materials. Note that the presence of the negative real part of permittivity that allows these polaritonic structures to be miniaturized essentially without limitation, and they will always resonate in the restrhalen bands. The elliptical resonator considered here has two main resonances, both are dipolar and lie in the RB2 band: one along the major axis and the other (at higher frequency) along the minor axis.

We consider an array of aligned h^11^BN ellipses on a CaF_2_ substrate so that each unit cell contains a single elliptical resonator (Figure 1a). Due to the interelement coupling, each original mode of the resonators generates a mode in the band structure of the array^43^ (Figure 1e). This is due to the fact that the resonant frequency depends on the phase difference ϕ between each resonator and its nearest neighbor. If the periodicity of the array is *P* then the phase difference can be associated with an in-plane wavevector *k* = ϕ/*P*. The band diagram simply relates ω to *k* for each mode.

A necessary (but not sufficient) condition for a mode to be bright is to be inside of the light cone delimited by  where *c* is the speed of light and *n* is the biggest among the refractive indexes of substrate and superstrate. Conversely, modes outside the light cone are always dark. In our case, because we work with polaritonic resonators that are deeply subwavelength, we will design structures with very small periodicity so it is easy to ensure that there are points in the band structure outside the light cone and, therefore, dark modes. Two notable points of the band structure are (Figure 1d,e):

- The Γ point for ϕ = 0, meaning *k* = 0, the center of the Brillouin zone, which can couple with normal incident light. This point is associated with the “bright mode” mentioned later.
- The *X* point for ϕ = π, meaning *k* = π/*P*, the dark mode at the edge of the Brillouin zone. We will refer to this mode as “dark mode” or BIC, as mentioned later.

We then progressively break the symmetry between adjacent ellipses by rotating them by a small angle θ in alternating directions (Figure 1b,c). Because of this, the new unit cell has a periodicity twice of the initial cell (*x*-periodicity *P*_*x*_ = 2*P*; *y*-periodicity *P*_*y*_). For this cell, the Brillouin zone is halved, and part of the band structure is folded, forming a new branch. The old dark mode *X* is renamed X′ (Figure 1e) and is folded at the Γ point and can now couple to normally incident light but notice that the polarization must be orthogonal compared to the corresponding bright mode, and this is because the dipolar momentum of the two resonators must be vectorially added together to determine the polarization of light that can interact with the mode. Because the dipoles are parallel in the bright mode but antiparallel in the quasi-dark, the results are two orthogonal polarizations for the two modes. The coupling strength increases monotonically with the magnitude of the perturbation so that the new mode is quasi-dark, i.e., quasi-BIC.

Coupling changes the band structure from a flat line and ultimately for the frequency shift between BIC and bright modes. This phenomenon occurs in general for any mode of the resonators. Since we focus here on the two aforementioned dipolar modes of the ellipses, we identify and study these 4 main modes out of 6 general modes (including dark modes **Du** and **Dv**) in the ellipse pair:

- **Bu**: Bright mode associated with major axis (u) mode.
- **Du**: Dark mode associated with major axis mode at θ = 0°.
- **Bv**: Bright mode associated with minor axis (v) mode.
- **Dv**: Dark mode associated with minor axis mode at θ = 0°.
- **qDu**: quasi-Dark or (q-BIC) mode associated with major axis mode.
- **qDv**: quasi-Dark or (q-BIC) mode associated with minor axis mode.

One of the key motivations to study these modes is the possibility of increasing the resonance quality factor due to the suppression of radiation losses, which are expected^44^ to be approximately proportional to θ^2^ and are studied in more detail in the next sections.

### Numerical Simulations

**Figure 2:**
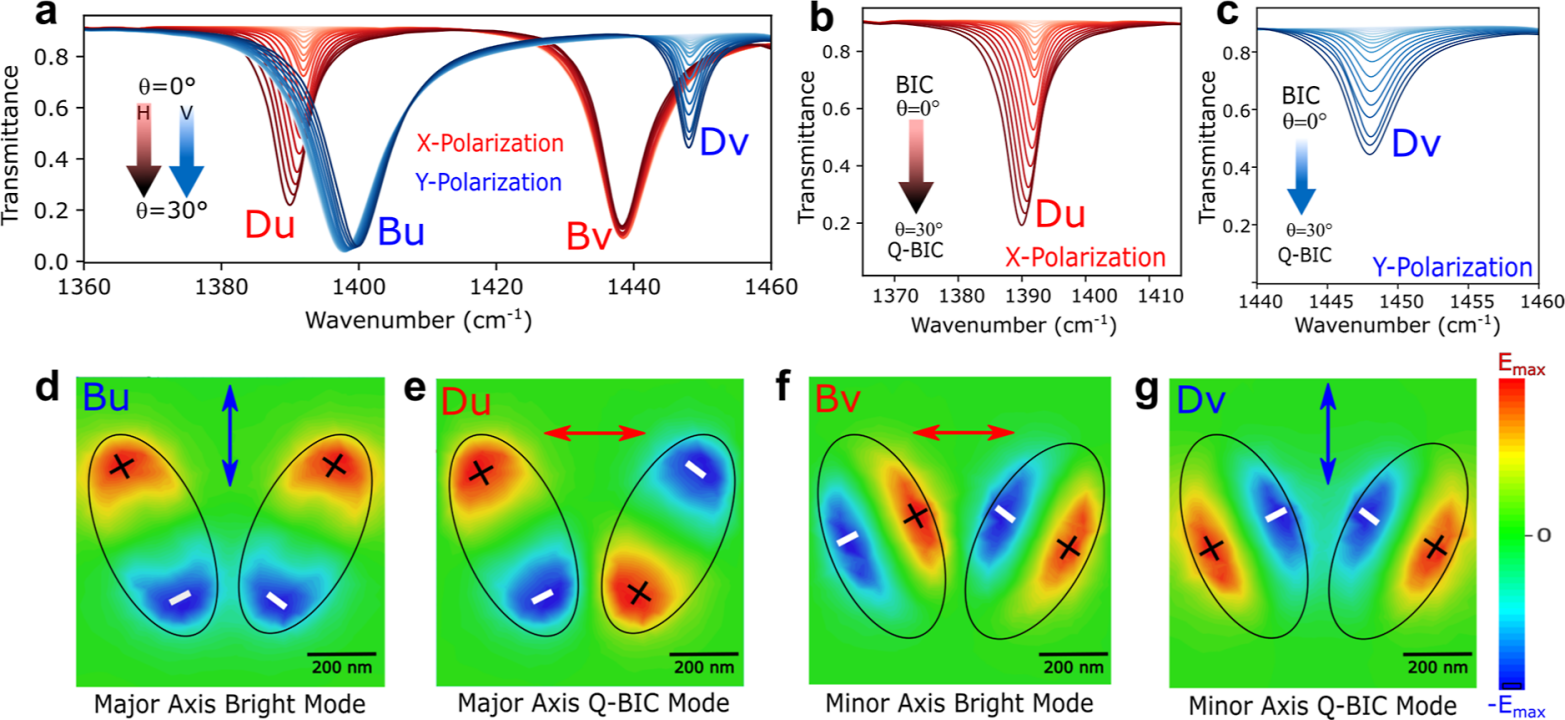
Simulations and electric field plots. (a) Numerically simulated transmission spectra for different values of the asymmetry parameter θ in the range 0 to 30° and for both incident polarization *X* and *Y*. (b,c) Magnification of (a) around the two q-BIC modes **qDu** and **qDv**. Unlike the bright modes (which are marginally affected by θ), the absorption dip of the BIC modes increases with increasing (θ) and vanishes for θ = 0. (d–g) Numerically simulated electric field plots (component *E*_*z*_ immediately above the resonators) with the same dimensions as Figure 1, for all the considered modes using an incident plane wave with polarization and frequency matching each of the targeted modes. The sign of the plot correlates with the sign of the induced charge density due to the ion core displacement and provides an excellent match to the theoretical model in Figure 1.

We numerically simulated the resonator to select the physical dimensions to be compatible with the requirements above, with the capabilities of our nanofabrication systems, and to show a significant resonant frequency separation to facilitate the experiment. We also simulate different sizes and periods of the resonator structure reported in Supporting Information (see Figures S2 and S3). Selected parameters of the structure with the dimensions above are mentioned in Figure 2. We simulated transmission for both incident polarizations along *X* and *Y* (Figure 2a–c) for values of the angle θ from 0 to 30°. Each of the four considered modes creates an absorption dip in the transmission spectrum. However, while bright modes are not significantly affected by the asymmetry parameter θ, the absorption dips of the q-BIC modes become deeper with larger θ, as expected due to the increased coupling with the far field.

Based on the preliminary analysis and experimental observations, a nominal rotation angle of θ = 25° was selected for the final experiments and will be employed throughout this study. This choice represents a compromise between the *Q* factor and the experimental amplitude of the peaks associated with the quasi-dark states. Specifically, larger angles yield more pronounced peaks but result in reduced *Q* factors. Initially, a numerical study was conducted, followed by an experiment with θ = 12°. However, the peaks at this angle were insufficiently resolved using our Fourier-transform infrared spectroscopy (FTIR) setup. Consequently, in the subsequent experimental phase, we opted for θ = 25°. This adjustment was made to enhance the peak intensity by approximately a factor of 4, thereby facilitating clearer resolution in our measurements.

Field plots (Figure 2d–g) are obtained by exciting the system at normal incidence with the polarization and frequency of each of the modes, and they match the theoretical model discussed earlier. For the long-axis modes, we find that the spatial distribution of the quasi-BIC mode is confined to a smaller region than the BICs in the symmetric system. In fact, Figure 2e shows that each negative pole of a dipole is close to the positive pole of the nearing dipole. As a result, the field is strong and confined between the two dipoles (as in a split dipole antenna) and weak in the rest of the region. In the bright mode the opposite happens: two dipoles are parallel and the 3D distribution of the field is more uniform. This explains the fact that the resonance frequency of that q-BIC mode is red-shifted for larger angles (Figure 2b).

**Figure 3:**
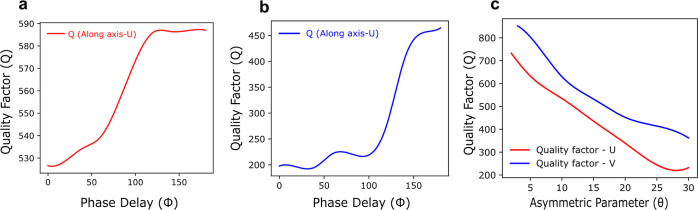
Quality factor simulations. (a,b) The simulated curve of phenomenological quality factor versus phase delay (ϕ) along *X* and *Y* polarization is represented in red and blue lines, respectively. The curves show two different regions: a ramp and then a plateau where *Q* saturates. We determined that the plateau starts in the regions outside the light cone. This is consistent with our model, which states that the radiative losses must vanish when the mode cannot radiate. The ramp region instead shows the worst *Q* factor at the center of the Brillouin zone, where radiation is more efficient. The value saturates at 590 and 450 in the two cases shown in the figures due to the polaritonic loss, and the radiative coupling begins at approximately (ϕ = 62°) (check Supporting Information). (c) Simulated *Q*-factor for the two modes of the single-ellipse cell as a function of the asymmetry parameter (θ). At θ = 0, the *Q*-factor is theoretically infinite, as confirmed by simulations. As θ increases, radiative losses cause the *Q*-factor to decrease. Conversely, as the mode approaches a Bound State in the Continuum (BIC) with a larger phase delay, the *Q*-factor increases.

The quality factor of the resonance is an important performance indicator of the final array. We first simulated the quality factor in the unperturbed symmetrical array (θ = 0) for the minor and major axes modes (along *x* and *y*, respectively) as a function of the phase delay between adjacent cells (Figure 3a,b). This simulation essentially maps the quality factor as a function of the in-plane momentum of the mode (See Supporting Information for more information). We also report the phenomenological *Q* factor in transmission (defined as the ratio of the resonant frequency to the full width at half-maximum in the transmission spectrum) as a function of the asymmetry parameter θ (Figure 3c). As expected, the *Q* factor is maximized for low values of the perturbation parameter θ. Notice that we are using here the following definitions for the *Q* factor. The regular *Q* factor is defined first by identifying the complex resonant frequency ω of the system, and its value is
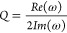


Experimentally, it is not always possible to access it, and therefore, another definition is commonly used, which we refer to as the “phenomenological *Q* factor”
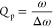
which corresponds to the more generally known definition of the ratio between resonant frequency and line width that can be extracted from a spectrum. There is a significant transition between the quality factor in Figure 3a,b because radiative losses are not possible for large phase delays because the modes are outside of the light cone. The two definitions of quality factor can be slightly different due to the saturation effect of the spectrum when the dip is close to the full extinction condition. A higher value of Q leads to a higher Purcell enhancement, which is known to be large in plasmonic systems. This is, however, true also of phonon polaritons due to the combination of very small modal volume and decent values of Q. For instance, Tamagnone et al.^5^ reported a Purcell factor enhancement of nearly 80,000, which is useful for applications involving light-matter interactions, such as sensing and photodetectors.^26^

**Figure 4:**
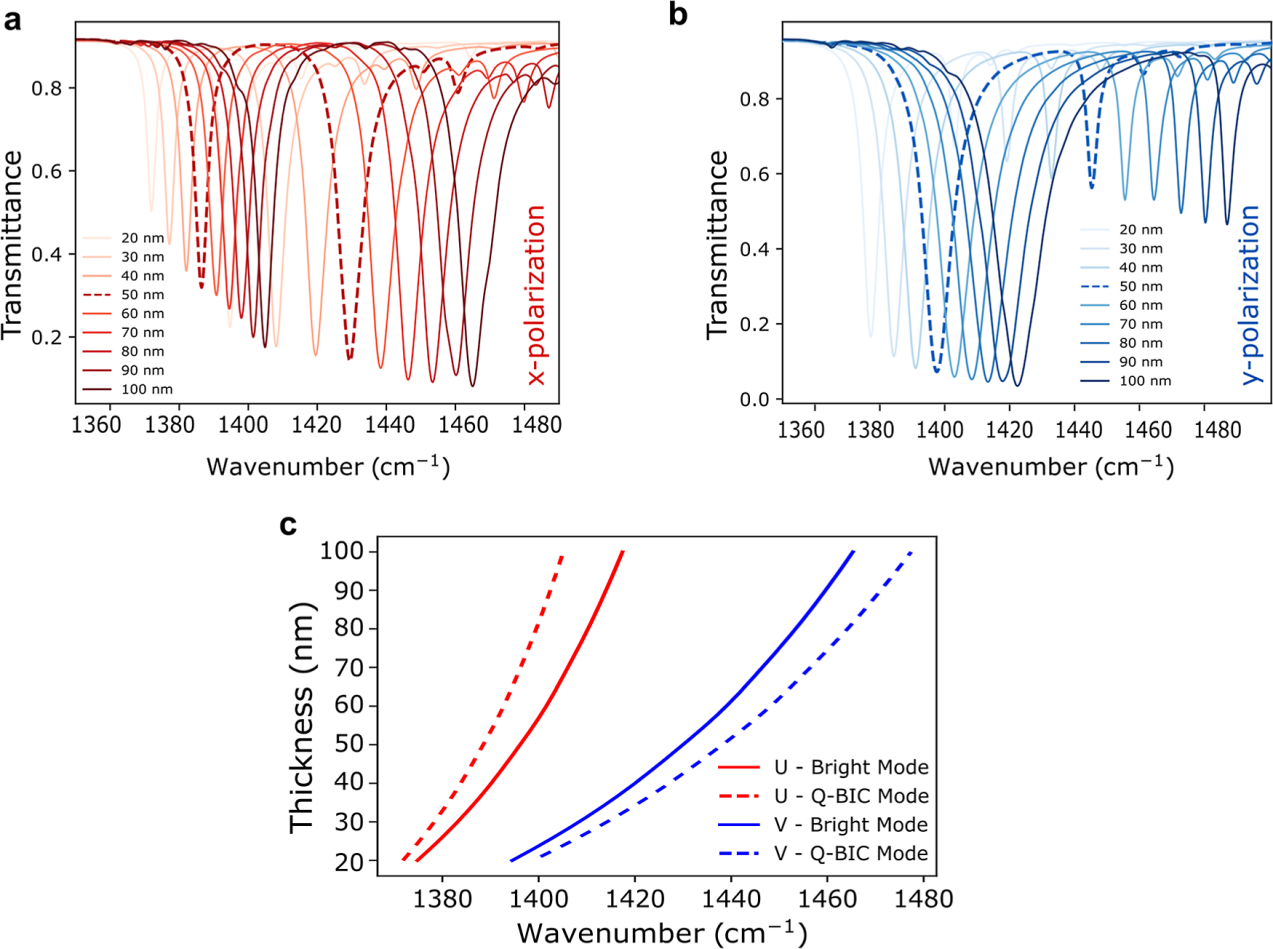
Thickness dependence and Q factor and of q-BIC states in hBN nanoresonator. (a,b) Simulated transmission spectra for both polarizations as a function of the thickness. Dashed lines mark the spectra in both plots for the target hBN thickness (50 nm). (c) Parametric simulations with thickness variation from 20 to 100 nm (steps of 10 nm) were performed, keeping θ = 25^°^ along both axis *U* and *V*. Variation in resonant frequencies as a function of the thickness of hBN resonators. A larger thickness leads to a blueshift in the resonances and to a larger separation between q-BIC and bright modes. This is due to increased coupling between resonators. Dependence of the *Q*-factor of the q-BIC state on the asymmetry parameter (θ) along *x* and *y*-polarization (red) and (blue), respectively.

The hBN thickness is also an important design parameter. Figure 4a–c illustrate the behavior of the system for different hBN thicknesses. Larger thicknesses lead to a blueshift of all resonances (Figure 4a,b) and, more importantly, to a larger separation between q-BIC and bright modes (Figure 4c). This is due to an increased coupling between resonators. Therefore, we selected an initial flake with a thickness of approximately 50 nm and adjusted the design of the resonators accordingly. We also reported all variations values with thickness in Supporting Information Table S1.

Finally, these simulations also allow the estimation of the spatial range of the coupling in the array, confirming that the structure exhibits long-range coupling. Long-range coupling is easier in dielectrics (and has been observed, for example, in many photonic crystal structures that show large sensitivity to the incidence angle), but it is challenging for deep subwavelength polaritons. The novelty here is that we resolve the angular dispersion of the modes, indicating that the resonators do not simply behave as an array of separate nanophotonic structures. Instead, collective modes are established, exhibiting a band structure. The fact that the resonance frequency of the dark states differs from that of the bright states clearly demonstrates that the resonators interact with each other. The simulations and theoretical analysis provided in the (Supporting Information) give a quantitative assessment of the interaction range, spanning approximately 12 cells.

It is well-known that long-range coupling is easier in dielectric resonators, and it has been observed in many photonic crystal structures that show a large sensitivity to the incidence angle of light.^45^ It has not been observed for phonon polariton resonators, where it is more challenging due to deep subwavelength resonances. In the Supporting Information, we show that the resonators do not simply behave as an array of separate nanophotonic structures. Instead, collective modes are established, and they exhibit a band structure. The fact that the resonance frequency of the dark states is different from the one of the bright states is alone a clear demonstration that the resonators are interacting with each other, but the simulation and the theory we provide in the Supporting Information give a quantitative answer to the range of the interaction, which spans multiple cells (approximately 12). This evaluation is based on the full-wave simulation, which takes into account the details of the shape of the resonators.

## Experimental Section

**Figure 5:**
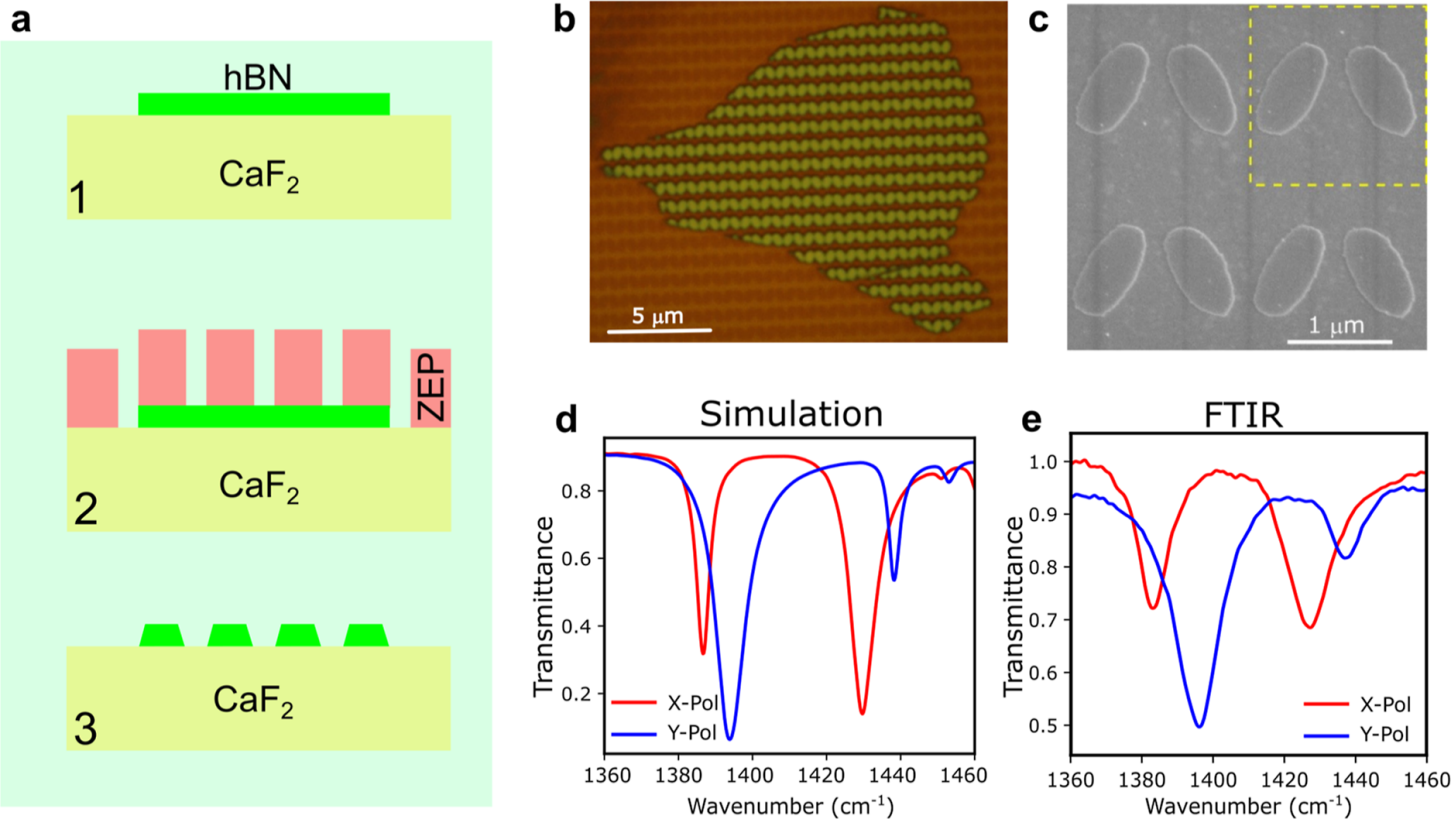
Schematic description of the nanofabrication and transmittance curve comparison. (a) Illustration of the nanofabrication of hBN elliptical resonators. (b) Optical microscope image of the hBN resonators after lithography. (c) Scanning electron microscopy imaging of the elliptical hBN resonators with θ = 25^°^ in which a yellow dashed square represents a unit cell of the array. (d–e) Comparison of numerical and experimental transmission spectra. (d) Shows the same curves as in Figure 2a for the case θ = 25^°^. The experimental curves in (e) were obtained using a polarization-resolved FTIR system coupled to a microscope along *x* and *y* polarization (red and blue) in color, respectively (See Methods).

The h^11^BN was first exfoliated by Scotch tape over a CaF_2_ substrate and then fabricated with a simple e-beam lithography step followed by reactive ion etching as explained in the Methods (Figure 5a–c).

To verify the presence of the q-BIC and bright modes, we measured the far field transmittance using a FITR (Fourier-transform infrared spectroscopy) system coupled with a microscope (Methods, Figure 5e).

The experimental FTIR curves (Figure 5e) match well with the simulations (Figure 5d), except for a broadening and shortening of the dips in the experiment due to imperfections and disorder in the fabricated resonators. The fact that the incident light is focused instead of an ideal plane wave also contributes to these nonidealities. Notice that the resonances are very clean and only appear for the correct polarization as predicted from the theory. This experiment already confirms that we successfully observed q-BIC states in a polaritonic system. Table 1 reports the measured resonances and compares them to the simulations at θ = 25° rotation of ellipses:

**Table 1:** Simulated and Experimental Resonances^a^

mode	freq (SIM), cm^–1^	freq (FTIR), cm^–1^	*Q*_P_ (SIM)	*Q*_P_ (FTIR)
**qDu**	1386.9	1383	322	184
**Bu**	1393.8	1396	160	121
**Bv**	1429.7	1427	204	135
**qDv**	1438.2	1437	423	204

a Modes are sorted by frequency. Resonance frequency and phenomenological quality factor *Q*_P_ are reported.

The table also compares the *Q* factor of the resonances for experiment and theory. In this case, we use the phenomenological quality factor Q_P_ defined as^28^
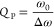
2where ω_0_ is the resonant frequency of the mode and Δω is the full width at half-maximum (fwhm) of the same mode evaluated from the transmission spectrum. Note that this quantity may be slightly affected by saturation phenomena for absorption lines that approach zero at their minimum. It is also important to note that the losses in hBN will limit the *Q* factor, but values close to the theoretical limit can be reached in this way, estimated here to be approximately 800 by eq 3(15)
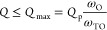
3

For the long-axis modes (**qDu**, **Bu**), the quality factor is enhanced 101% numerically and 52% experimentally with respect to bright modes, while for the short-axis ones (**qDv**, **Bv**), the quality factor is enhanced 107% numerically and 51% experimentally with respect to bright modes. These figures confirm the enhancement discussed above.

The quality factor enhancement is also related to an improved lifetime τ of the polaritonic mode as the relationship between lifetime and *Q* factor can be expressed mathematically as
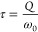
3a

The larger value of the quality factor, and therefore a larger lifetime of the polariton, allows for a prolonged light-matter interaction and higher field enhancement, which can increase the performance of devices such as photodetector and biosensing and can potentially find application also for nonlinear optics.

**Figure 6:**
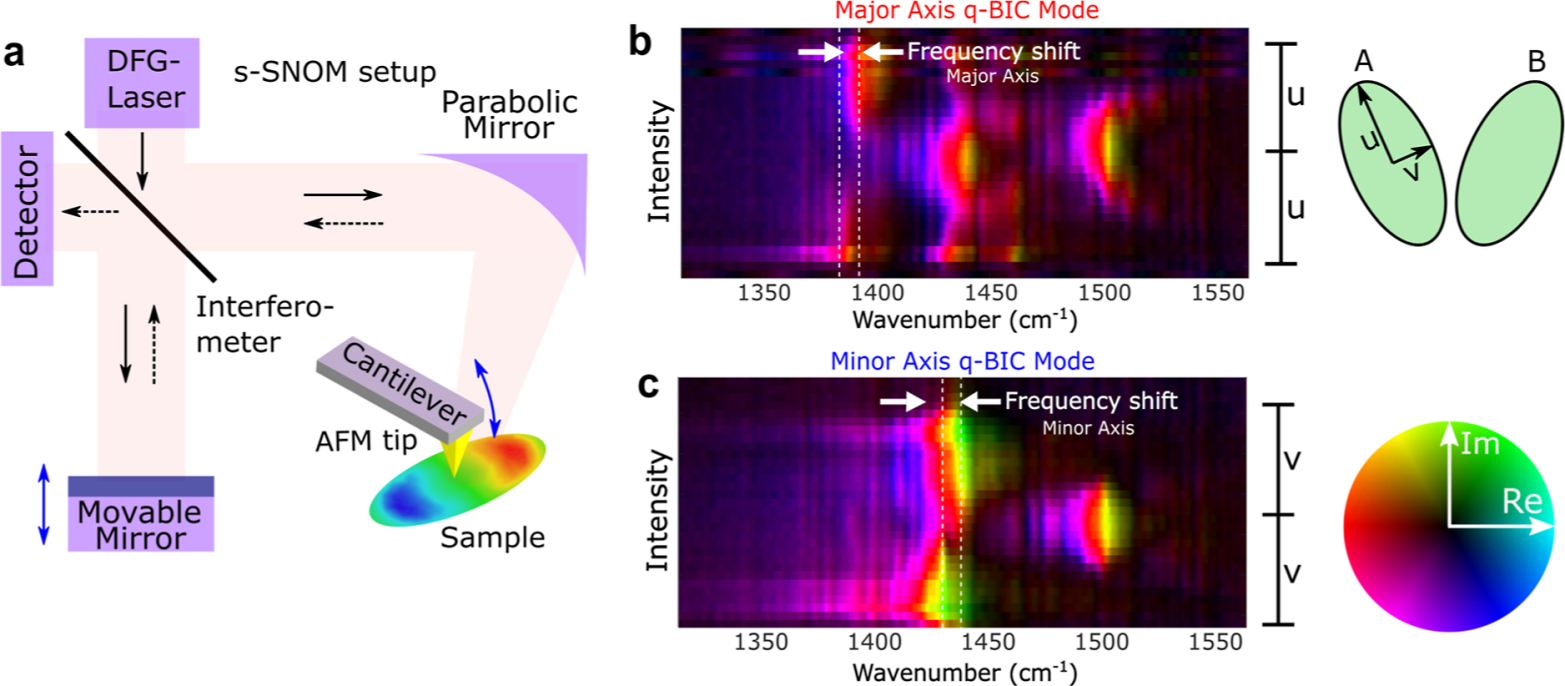
NSOM characterization. (a) Schematic of the used nano-FTIR setup. A Michelson interferometer is used to retrieve the amplitude and phase of the scattered near-field signal. The system uses a DFG source which guarantees at the same time large spatial coherence and small temporal coherence (resulting in a wideband spectrum). (b) Nano-FTIR spectra collected as a function of the position of the tip along the major axis of one of the resonators (A), with the polarization of light selected to excite the related q-BIC mode (here, the **Du** mode of Figure 1d). The bright mode is also visible because it has a much stronger coupling with light than the q-BIC one. This allows us to measure the shift between the modes (9 and 8 cm^–1^ for major and minor axes, respectively, in good agreement with far-field measurements and simulations). Other higher-order modes are also visible at larger wavenumbers. Color bar: the color represents the phase (from 0 to 360°), brightness, and amplitude (normalized to 1). (c) Same as (b) but for the q-BIC of the minor axis (**Dv** in Figure 1d). To the right of the spectra in (b,c), we reported the sizes of the ellipse’s axes for comparison (See Supporting Information S2).

The far-field measurements already prove clearly the experimental observation of the BIC states but do not offer a direct validation concerning the geometry of the modes. To obtain this validation, we used a nanoscale version of FTIR (nano-FTIR) based on a scanning near-field optical microscope (SNOM) of a commercial Neaspec system (Figure 6a). During these measurements, a broadband mid-IR laser was focused under a sharp AFM tip. The tip was scanned with respect to the sample surface, and the scattered light was collected by a detector, whose signal (after demodulation with tip oscillations see Methods) represents the local near-field of the resonator with a spatial resolution of the order of the tip radius (about 50 nm). An interferometric setup allows the retrieval of the complex spectrum, as in Figure 6b,c, and hyperspectral imaging video (see Supporting Information).

Figure 6b,c represents the near field response as a function of the position on the axes of the ellipses and the wavenumber. For these experiments, we first aligned the sample in such a way that the polarization of the incident light would couple with one target q-BIC mode (“**qDu**” and “**qDv**” in Figure 6b,c, respectively). We then collected spectra while scanning the tip along the corresponding axis of one of the ellipses (major axis for Figure 6b, minor axis for Figure 6c). Thanks to this arrangement, we see the q-BIC mode, but at the same time, we also see a portion of the bright mode. This is due to the fact that the bright mode has a much stronger coupling and can be excited due to the experimental imperfections of the system. Incidentally, this allows us to directly compare in a single experiment both the q-BIC and the corresponding bright mode, hence their frequency separation (approximately 10 cm^–1^, which matches Table 1).

SNOM images of polaritonic are not, in general, easy to interpret due to the complexity of the coupling phenomena that occur in the tip–sample system. In particular, several scattering processes can occur, and they interfere to form the final images which are the result of a superposition of multiple contributions. Each contribution is due to a different process. In the work by Tamagnone et al.,^5^ we detailed the most important processes in the case of hBN resonators:

- Local tip–material interaction
- Direct excitation of a bright mode and tip read-out (referred to as direct contribution)
- Excitation of a mode (bright or dark) by the tip and tip read-out (referred to as round-trip contribution)

For simple systems (e.g., a disc resonator) it is possible to extract the contributions and modes exploiting the symmetries. But in this case, the system is much more complicated. Nevertheless, the NSOM scans are important because they allow the verification of the local geometry of the modes. We note that bright and dark states appear in different regions of the resonators (Figure 6c). The same was observed for dipole antennas.^5^

The explanation is that the same mode contributes to the image both via the round-trip contribution and the direct contribution. Because the amplitude of the round trip contribution is approximately the square of the direct one, the contributions interfere constructively on one side of the antenna and destructively on the other.

In this case, the opposition in phase in the dipolar modes causes the bright mode to be visible on one of the halves of the antenna and the BIC mode to be visible on the other half. In addition, modes on the major and minor axes have the expected frequencies and are consistent with the selected light polarization. In addition, modes on the major and minor axes have the expected frequencies and are consistent with the selected light polarization.

## Methods

### Theoretical Simulations

Simulated transmittance spectra of the hBN resonators array were obtained using Ansys HFSS finite element method electromagnetic solver. The software was used to calculate modes and scattering parameters (S-parameters) of the structures in the frequency domain. The resonator was excited using a port, and a frequency sweep was performed in the range of 1300 to 1500 cm^–1^ in the mid-IR region.

### Metasurface Nanofabrication

Hexagonal boron nitride crystals were grown via precipitation from a Fe solvent in an N_2_/H_2_ atmosphere using isotopically enriched (>99%) elemental ^11^B powder as the boron source material. As the source of nitrogen, we used naturally abundant gas, which is 99.6% ^14^N and 0.4% ^15^N.

These h^11^BN crystals were mechanically exfoliated via the scotch tape method on a 5 mm thick CaF_2_ substrate. Suitable flakes were identified using an optical microscope, and their thicknesses were measured with an atomic force microscopy (AFM-Park XE-100). Afterward, a layer of the positive-tone electron beam resist ZEP-520A (2:1 of Anisole: ZEP-520A) was spun onto the CaF_2_ substrate, baked at 90 °C for 180 s and then at 180 °C for 180 s. An electrically conductive layer of 10 nm of gold was sputtered with a Kenosistec KS500 confocal sputter coater system on the top of the resist. The patterns were defined using an electron beam lithography system (Raith-150 Two) with an acceleration voltage of 30 kV, an aperture size of 20 μm, and an area dose of 90 μC/cm^2^. After the lithography, the conductive gold layer was etched with a commercial gold etchant for 10 s, and then the resist was developed in the ZEP Developer at 5 °C for 60s with a subsequent 30 s rinse in IPA. The patterns were verified with an optical microscope. hBN was then etched with an inductively coupled plasma-reactive ion etching (ICP-RIE) with ZEP as a masking layer. ICP-RIE was performed for 80 s using 10 sccm of CHF_3_ under the pressure of 5 mTorr with 60 W of RF power and 450 W of ICP power. Finally, the ZEP mask was stripped by placing the sample in remover-PG at 80 °C followed by rinsing in the DI water.

### Measurements

The far-field optical characterization of the hBN resonators was performed using a commercial Fourier transform infrared spectrometer (Thermofisher iS50 FTIR). Transmittance and reflectance were measured in the range 1300–1500 cm^–1^ with an illumination aperture of 20 μm. A holographic polarizer was added in front of the detector for polarization-resolved measurements.

Near-field characterization was performed with a commercial Neaspec system using the related nano-FTIR module with a broadband spatially coherent DFG (difference frequency generation) mid-IR source and a Michelson interferometer using the full travel range of the mirror (1.5 mm, corresponding to a 3 cm^–1^ spectral resolution). Demodulation of the tip–sample scattered signal with a lock-in set at two times the frequency of the AFM tip is performed to select the near-field signal coming from the pure tip–sample interaction (hence, reaching a spatial resolution compared to the tip radius, about 50 nm). Due to the geometry of the interferometer, it is possible to retrieve both the amplitude and the phase of the scattered near-field. A systematic offset of 14 cm^–1^ was observed with respect to the expected response of the materials and the FTIR measurement. We also experienced this offset while we were measuring known reference spectra. In particular, we selectively operated with single wavelengths of a CW Ti:Sa laser, tunable in the 700–1000 nm range, and collected the reflected light when the tip (silicon with a Pt–Ir coating) was on top of a silicon substrate. The shift at all wavelengths oscillates between 0.5 to 1% of the measurement wavelength. All measurements presented here have been corrected to take into account this offset. Hyperspectral maps with phase and amplitude were obtained by scanning along the axes of the ellipses and in a raster grid.

## Conclusions

Our demonstration of BIC and q-BIC states in a polaritonic system paves the way for several new research directions. Since polaritonic resonators can be miniaturized indefinitely (except ofcourse for the atomic limit), it is possible to create very complex unit cells (even with more than two nanostructures) with a very rich spectrum of BIC and dark modes. There are many ways to generalize these results, such as creating more complex patterns where one or more perturbations are introduced along all the dimensions of the array. In all cases, the perturbations can be tuned to control the amount of coupling of these modes with the environment (through the radiative channels), creating interesting trade-offs also for light manipulation, optoelectronics, and metasurfaces, besides the obvious applications for sensing and detection. Exploring different materials, such as excitonic and plasmonic monolayers, will make this platform even more flexible thanks to the possibility of tuning these materials electrically.

## Data Availability

The code that supports the findings of this study is available from the corresponding authors upon reasonable request

## Supporting Information Available

A hyperspectral imaging video as S2(.mp4) is attached with Supporting Information which represents the availability of dark and bright modes along both axis of the ellipses (MP4).
Figure S1 shows the comparison of the True BIC and Q-BIC states by breaking the symmetry of the system. Figure S2 shows simulation transmission spectra for the different resonator sizes. Figure S3 shows simulation transmission spectra for the different period sizes. Table S1 represents the thickness dependence of hBN resonators in Q-BIC states. Theory and calculations regarding the long-range coupling of polaritons in hexagonal boron nitride nanoresonators. Phase Delay calculations are also provided. Simulation and optimization of the structures are also provided in the Supporting Information (PDF).
