# Supplementary material for: Bound States in the Continuum and Long-Range Coupling of Polaritons in Hexagonal Boron Nitride Nanoresonators
Source: ACS Photonics. 2024 Sep 22;11(10):4017–26. doi: 10.1021/acsphotonics.4c00358 (PMC11487684; doi:10.1021/acsphotonics.4c00358)
Supplement: Supplementary file 2 — ph4c00358_si_002.pdf [file ph4c00358_si_002.pdf]

Supporting Information

Bound states in the continuum and long-range coupling of polaritons in hexagonal boron nitride nanoresonators

Harsh Gupta[1, 2, *], Giacomo Venturi[3, 4], Tatiana Contino[1,2], Eli Janzen[5], James H. Edgar[5], Francesco De Angelis[1], Andrea Toma[1], Antonio Ambrosio[3], Michele Tamagnone[1, *]

1) Istituto Italiano di Tecnologia, via Morego 30, 16163 Genova, Italy

2) Dipartimento di Chimica e Chimica Industriale, Università degli Studi di Genova, via Balbi 5, 16126 Genova, Italy

3) Center for Nano Science and Technology, Fondazione Istituto Italiano di Tecnologia, Milan, Italy

4) Dipartimento di Fisica, Politecnico Milano, Piazza Leonardo Da Vinci 32, Milano 20133, Italy

5) Tim Taylor Department of Chemical Engineering, Kansas State University, Manhattan, KS, 66506, USA

* Corresponding Authors harsh.gupta@iit.it, michele.tamagnone@iit.it

Comparison of perturbed and unperturbed systems:

In Figure S1, we present the comparison of simulated transmittance spectra between two configurations of the elliptical hBN resonators, considering both axis major (u) and minor (v) correspond to the X and Y polarizations, respectively. In the first configuration (Figure S1a), where the symmetry is protected by no rotation of the ellipses, the transmission spectra reveal the presence of bright modes at 1393.8 cm^{-1} and 1429.70 cm^{-1} along axes u and v, respectively. These bright modes exhibit peaks in the transmission curves, indicating the resonant behavior of the hBN in the upper restrahlen band. At zero-degree rotation, there are no channels to radiate these structures since they are symmetry-protected. It gives us theoretically infinitely high-quality factors (FWHM nearly zero) such as quality factor defined by eq. S1, where Q is the quality factor and Δf is the FWHM of the resonance.

$$Q = \frac{\omega_0}{\Delta\omega} = \frac{f_0}{\Delta f} \qquad \text{(Eq. S1)}$$

However, an important phenomenon occurs after introducing a rotation of 25 degrees to the elliptical resonators in the second configuration, shown in Figure S1b. While the bright modes persist in both axis modes along both polarizations, additional modes emerge with a distinct behavior known as the quasi-bound state in the continuum (qBIC) mode. These qBIC modes are at 1386.9 cm^{-1} and 1438.20 cm^{-1} as qDu and qDv along x and y polarizations, respectively, and are characterized by dips in the transmission curves.

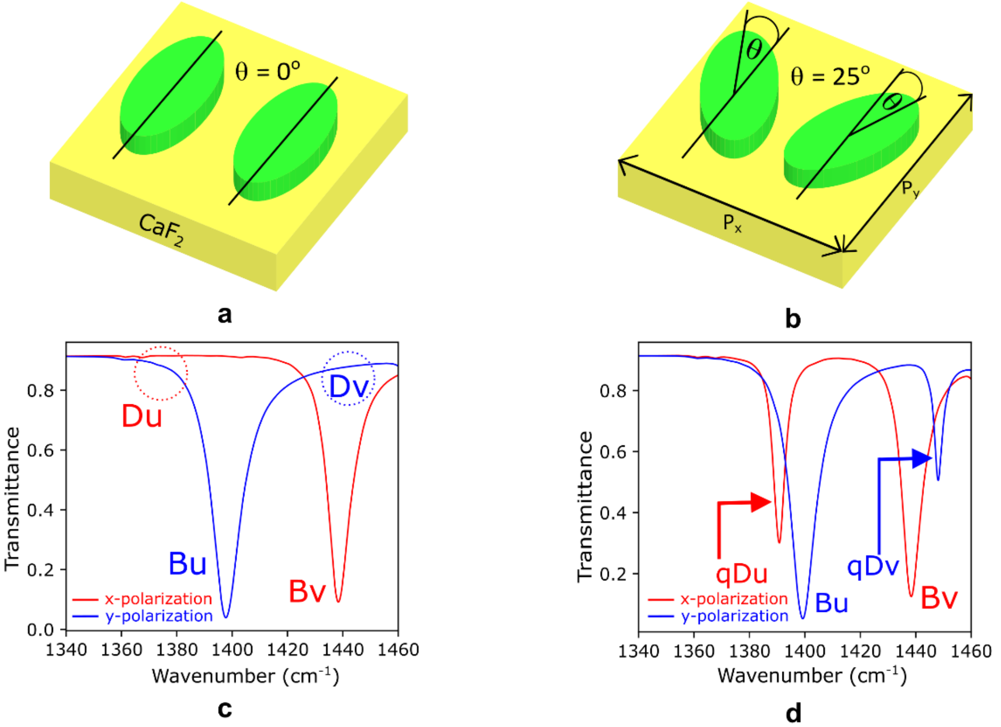

Figure S1. Appearance of Q-BIC modes. (a) Two symmetrical elliptical hBN resonators are placed on Caf_2 with 0° rotation. **(b)** Both resonators are now rotated by 25 degrees along their x-y plane. **(c)** Here, the spectra represent the bright modes along the u (major) and v(minor) axis named as Bu and Bv. Du and Dv dark modes along the major and minor axis, respectively which are non-radiating in nature, we need to break their symmetry to make it radiative. These modes are infinitely high-quality factors such as zero FWHM represented by a dotted circle. **(d)** Transmission spectra represent the presence of qBIC modes as qDu and qDv along the major and minor axis, respectively, along their bright modes, arising after breaking the symmetry through the 25-degree rotation along their plane.

Simulation and optimization of the structures

We use ANSYS HFSS for our simulations, which is Maxwell's equations solver. The software allows to specify a static system defined by several geometrical regions, each with their own constitutive equations (in this case simply a different dielectric tensor for each region). The software uses an adaptive finite elements mesh, and convergence is ensured by comparing the results of consecutive iterations

We optimized the various parameters of the unit cell in the following order:

1. The overall periodicity of the array was chosen so that the structures would be clearly sub-wavelength. The periodicity in our case is of 0.9 um along one direction and 1.8 um in the other, while the operation wavelength is about 7 um. The period is a free choice given that the polariton can be miniaturized significantly, but clearly, if the structures are too small, then the fabrication becomes challenging.
2. The aspect ratio of the ellipses was set to 0.5 to ensure that the modes along the major and minor axis would be well separated in frequency. This was verified with an early simulation

3. The hBN thickness proved to be a very important parameter: sweeps of the thickness indicated that the separation of the bright and quasi-dark modes strongly depends on the thickness. For larger thickness the separation is larger, and therefore we opted for 50 nm, which is a trade-off with the reactive ion etch capabilities available to us.
4. The angle was chosen to ensure that the bright mode would not completely hide the quasi-dark mode. Here, too, a sweep was used to determine the best trade-off. we first did a numerical study and performed a first experiment with θ = 12°. For this angle, the peaks were not strong enough to be resolved with our FTIR, so in the second experimental batch, we used θ = 25°. This final value was chosen to increase the size of the peak by approximately a factor of 4.
5. Finally, it is worth mentioning that the simulation of hyperbolic modes can be quite difficult because the wave vector inside the material can have very large values. To ensure convergence, the software used (Ansys HFSS) implements adaptive meshing: the mesh is progressively improved in the regions with large electric fields, and a convergence criterion based on the comparison or subsequent iterations is used to ensure that each simulations are converged.

Thickness dependence on the modes:

We also present here our simulation study, where we explored the impact of hBN layer thickness variation on the resonant frequencies of the bright and quasi-bound states in the continuum (qBIC) modes in the 25-degree rotated configuration. We systematically varied the hBN thickness from 20 to 100 nm with a step size of 10 nm and examined the corresponding shifts in the resonant frequencies plotted in the article and illustrated in Table S1. Theory explained in the article.

#	Thickness hBN (nm)	Major Axis-(u) Bright Mode Frequency (cm^{-1})	Major Axis-(u) qBIC Modes Frequency (cm^{-1})	Minor Axis-(v) Bright Mode Frequency (cm^{-1})	Minor Axis-(v) qBIC Modes Frequency (cm^{-1})
1	20	1374.80	1371.80	1394.50	1398.80
2	30	1382.60	1376.85	1407.50	1414.20
3	40	1388.25	1381.85	1419.50	1427.10
4	50	1393.85	1386.95	1429.70	1438.20
5	60	1399.20	1390.72	1438.20	1448.20
6	70	1404.20	1394.50	1446.20	1456.50
7	80	1408.60	1398.0	1453.20	1464.20
8	90	1413.10	1401.30	1460.0	1471.10
9	100	1417.40	1405.10	1465.30	1477.40

Table S1 Variation of modes with hBN thickness. Variation of resonant frequencies of Bright and qBIC modes along their major(u) and minor(v) axis with changing the thickness of hBN.

Resonator Sizes and Periodic dependence on the modes:

Besides measurements of the effect of thickness in the qBIC modes, we also measure how the qBIC and bright modes vary by changing the resonator's aspect ratios and the period sizes. Firstly, we made the variation by fixing the major axis (u) and made variations only along the minor axis diameter (2v) from 414 - 714nm with a 20nm step size. Variations along the minor axes cause more interaction along the minor axis modes. Hence, the resonances shift due to their different sizes, as shown in Figure S2(b-c). Secondly, we made the variation by fixing the minor axis (b) and made variations only along the major axis diameter (2u) from 925 to 1125nm with a 20nm step size. Here, we also get a similar behavior as we expected; the resonances shifted only along the major axis, as the minor axis was fixed.

All the resonances described above are red-shifted, as expected, due to the fact that larger resonators tend to have lower natural frequencies. The resonance frequency for a specific mode in a resonator is determined by the speed of the wave in the material and the physical dimensions of the resonator. For instance, in a one-dimensional resonator (like a string), the fundamental resonance frequency is given by equation S2. *Ref (1-2)*

$$f \approx \frac{v}{2L} \qquad \text{(Eq. S2)}$$

Where v is the wave speed in the material, and L is the length of the resonator. Here in our elliptical structures, the factors that are dependent on the resonances are *u* and *v*, so the phenomenon should be the same, but the frequency now is nearly dependent on the equation S3

$$f \approx \frac{c}{2\pi} \sqrt{\left(\left(\frac{m}{u}\right)^2 + \left(\frac{n}{v}\right)^2\right)} \qquad \text{(Eq. S3)}$$

Where c is the speed of electromagnetic waves in the material, a is the length of the major axis, b is the length of the minor axis, m and n are mode numbers (integers) corresponding to the number of half-wavelengths fitting along the major and minor axes, respectively.

We change the dimension of the resonator one at a time such that firstly, the major axis *a* is fixed and the minor axis *v* is varying, and correspondingly, we get red-shifted resonances(bright and qBIC) by (eq S3) as minor axis *v* increases, shown in Figure S2 (b-c), Here the mode depends only on minor axis *v*,

$$f \approx \frac{v}{2v} \qquad \text{(Eq. S4)}$$

Similarly, the same phenomenon can be seen when the minor axis *b* is fixed and the major axis *u* is varying, corresponding with eq. S4, all the resonances (bright and qBIC) are red-shifted by increasing the sizes along the major axis *u* by eq. S4.

$$f \approx \frac{v}{2u} \quad \text{(Eq. S5)}$$

We also simulate the variation of the resonant modes with the period sizes, as shown in Figure S3. Transmission spectra show that changing the period size (distance between the centers of adjacent resonators) can affect the coupling between individual resonators. If the period is decreased (resonators are brought closer together), the coupling between them typically increases, which can alter the individual and collective resonance frequencies. As the distance between resonators increases, the coupling between them generally weakens. This weakening of coupling typically results in a red shift in the resonance frequency due to a decrease in interaction energy. Consequently, the resonators start behaving more like isolated entities, and their resonance frequencies tend to be lower compared to when they are strongly coupled and close together.

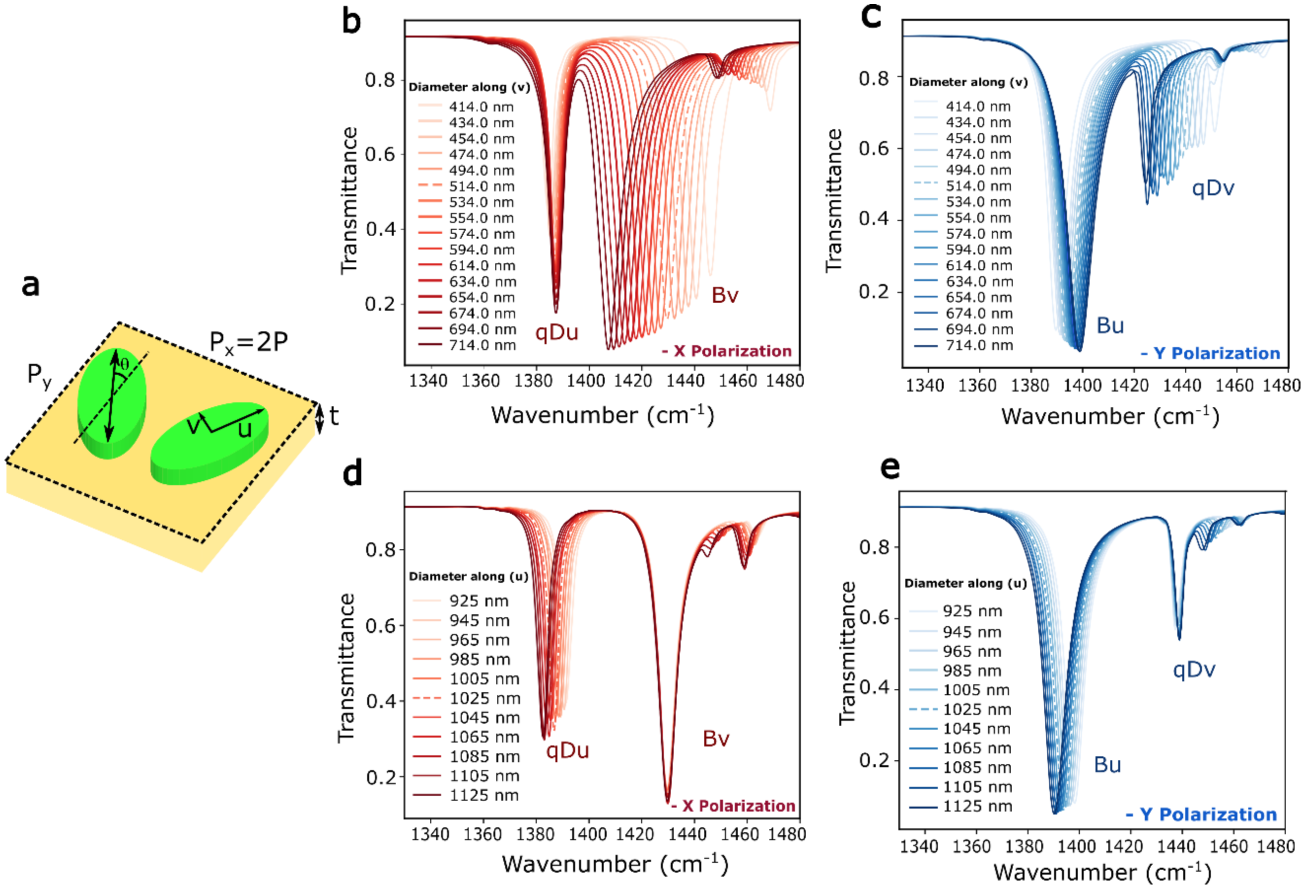

Figure S2. Simulations Transmission spectra for the different resonator sizes. (a) Structure for the simulations where *u* and *v* are the major and minor axis, respectively, and P is the period size. **(b)** Transmission spectra show the resonances qBIC (qDu)

and bright mode (Bv) in x polarization. Major axis *u* is fixed, and variation occurs only along minor axis *v*, such that only minor axis modes have redshifts with their sizes. As the size along *v* increases, there is a redshift of frequency. Dotted curves shows the parameter we used in the main experiment and simulations. **(c)** Transmission spectra show the resonances qBIC (qDv) and bright mode (Bu) in y polarization, where the same phenomenon is confirmed as point (b). **(d)** Transmission spectra show the resonances qBIC (qDu) and bright mode (Bv) in x polarization. Minor axis *v* is fixed, and variation occurs only along major axis *(u)* so that only major axis red-shifted modes with their increased sizes can be seen. As the size along the major axis *(u)* increases, there is again a redshift of major axis frequency modes. **(e)** Transmission spectra show the resonances qBIC (qDv) and bright mode (Ba) in y polarization.

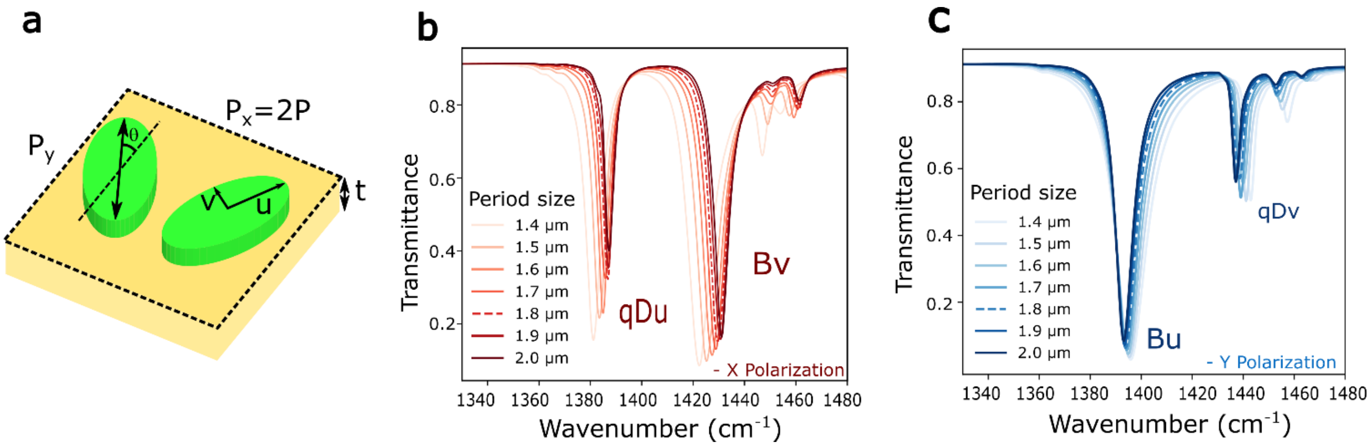

Figure S3. Simulations Transmission spectra for the different period sizes. (a) Structure for the simulations where *u* and *v* are the major and minor axis, respectively, and P is the period size. **(b)** Transmission spectra show the resonances qBIC (qDu) and bright mode (Bv) in x polarization. It also implies that as the period p increases, the resonant frequency (ω) decreases, which is also suggested by the dispersion relation in a periodic system. The period size varies from 1.4µm to 2.0µm, where the dotted curve represents the 1.8µm period size that we used in our experiments. **(c)** Transmission spectra show the resonances qBIC (qDv) and bright mode (Bu) in y polarization.

Theory of Long-range interactions:

The studied interaction is long-range, as it spans approximately **12** consecutive elliptical resonators. The method used to estimate this number is presented here.

Let us explicitly name the band structure of the considered mode as

$$\omega = f(k) \quad ; \quad k = g(\omega)$$

where *f* and *g* are complex analytical functions and are inverse of each other. The group and phase velocities are given by:

$$v_G = \frac{\partial \omega}{\partial k} = f'(k) = \frac{1}{g'(\omega)}$$

$$v_P = \frac{\omega}{k} = \frac{f(k)}{k} = \frac{\omega}{g(\omega)}$$

We can consider two different types of situations:

1) The structure is excited with a given *k* (real) at an initial time, and the oscillations will dampen in time. The dampening is associated with a complex frequency and temporal quality factor Q_T:

$$Q_T = \frac{\mathrm{Re}(\omega)}{2\,\mathrm{Im}(\omega)}$$

2) The structure is excited with a given ω (real) at an initial position, and the oscillations will dampen as they propagate in time. The dampening is associated with a complex momentum and spatial quality factor:

$$Q_S = -\frac{\mathrm{Re}(k)}{2\,\mathrm{Im}(k)}$$

The numerical simulations estimated Q_T in the range 200 to 600, with an average of 400. Using case 1, the large value of Q_T allows this approximation:

$$\omega = e^{i\phi}|\omega| \cong (1 + i\phi)|\omega| = \left(1 + \frac{i}{2Q_T}\right)|\omega|$$

A Taylor expansion of *g* gives:

$$g(\omega + d\omega) \cong g(\omega) + \frac{1}{v_G}\,d\omega$$

It follows, for any real k_R:

$$k_R = g(\omega) \cong g\left(\left(1 + \frac{i}{2Q_T}\right)|\omega|\right) = g\left(|\omega| + \frac{i|\omega|}{2Q_T}\right) \cong g(|\omega|)$$

$$g(|\omega|) = k_R - \frac{i|\omega|}{2v_G Q_T}$$

This allows us to bridge case 1) with case 2). Considering, in fact a real ω, we get $\omega = |\omega|$ and:

$$g(|\omega|) = k_R - \frac{i|\omega|}{2v_G Q_T}$$

The spatial quality factor is then given by:

$$Q_S = \frac{k_R}{\frac{|\omega|}{v_G Q_T}} \cong \frac{v_G}{v_P} Q_T$$

The relationship

$$Q_S \cong \frac{v_G}{v_P} Q_T$$

is very fundamental and allows to calculate the spatial quality factor from the temporal one. The range of the spatial interaction is then given by the product of the quality factor and the spatial period:

$$R = \frac{Q_S}{4\pi/k}$$

Evaluating this expression with the numerical results provides a range of approximately 12 cells.

Phase Delay Calculations

Explains the phase delay calculations for the plot of Figure 3 of the Research article. The exact value of the phase delay for which radiative coupling begins can be estimated from the light cone dispersion:

$$\omega = \frac{c}{n} k_x$$

As the reviewer suggests, we can relate the phase difference to the k vector:

$$k_x = \frac{\phi}{P}$$

Replacing the cell parameters and using the relation

$$\omega_0 = \frac{2\pi c}{\lambda}$$

we obtain:

$$\phi = 2\pi n \frac{P}{\lambda}$$

Which gives a value of 62° by putting the corresponding values of the parameters according to the simulations.

Hyperspectral Imaging

For better understanding and in support of the findings presented in this paper, we provide supplementary material featuring a Near-Field Scanning Optical Microscopy (NSOM) hyperspectral imaging video. This video offers a dynamic visualization (for various frequencies) of the details captured during the measurement. The NSOM hyperspectral imaging technique allows imaging of both bright and dark modes in the array. Importantly, the video shows both the phase and the amplitude of the modes imaged. Each frame corresponds to a frequency in the hyperspectral cube.

References

1. Cahyadi, Danang D., et al. "Experimental study of resonance frequency at prime mover thermoacoustic standing wave." Journal of Physics: Theories and Applications 1.2 (2017): 157-166.
2. Bahaa, E. "Saleh; Malvin, Carl Teich." Fundamentals of Photonics, 2nd Edition: Wiley (2007).
